# Brain imaging screening in metastatic breast cancer: patients**’** and physicians**’** perspectives

**DOI:** 10.1016/j.breast.2025.104558

**Published:** 2025-08-12

**Authors:** Leonor Matos, Mette van Ramshorst, Volkmar Müller, Elisa Agostinetto, Sabine Linn, Matteo Lambertini, Veronique Dieras, Fanny le Du, Sofia Braga, Carmen Criscitiello, Katarzyna J. Jerzak, Gil Morgan, Sara Brucker, Patricia von Kroge, Renate Haidinger, Gema Rodríguez Recio, Eva Schumacher-Wulf, Mario Fontes Sousa, Francesco Schettini, Elena Laakmann

**Affiliations:** a Breast Unit, Champalimaud Clinical Center, Champalimaud Foundation, Lisbon, Portugal; b Netherlands Cancer Institute, Amsterdam, the Netherlands; c University Medical Center Hamburg-Eppendorf, Germany; d Université libre de Bruxelles (ULB), Hôpital Universitaire de Bruxelles (H.U.B), Institut Jules Bordet, Service de Oncologie, Rue Meylemeersch 90, 1070, Bruxelles, Belgium; e University of Genova, Italy; f IRCCS Ospedale Policlinico San Martino, Genova, Italy; g Department of Medical Oncology at Centre Eugene Marquis, Rennes, France; h Institut José de Mello Saúde, Lisbon, Portugal; i European Institute of Oncology, Milan, Italy; j Sunnybrook Odette Cancer Centre, University of Toronto, Toronto, Canada; k Skåne University Hospital, Lund, Sweden; l Department for Women's Health, University Medical Center, Tübingen, Germany; m Brustkrebs Deutschland e.V., Hohenbrunn, Germany; n Asociación Española Cancer de Mama Metastásico, Madrid, Spain; o Mamma Mia! Magazine, Cologne, Germany; p CUF - Hospitais e Clínicas, Lisbon, Portugal; q Unidade Local de Saúde Lisboa Ocidental, Lisboa, Portugal; r Medical Oncology Department, Hospital Clinic of Barcelona, Barcelona, Spain; s Faculty of Medicine and Health Sciences, University of Barcelona, Barcelona, Spain

**Keywords:** Brain metastasis, Metastatic breast cancer, Survey, Brain imaging, Screening

## Abstract

**Background:**

Routine brain imaging screening (BIS) in patients with metastatic breast cancer (BC) without neurological symptoms is currently not recommended, as no survival/quality-of-life improvements have been demonstrated. We aimed to examine physicians and patients’ attitudes and perceptions toward BIS.

**Methods:**

International cross-sectional online survey for patients and physicians, distributed from May 2023 to February 2024. Patients with BC diagnosis were deemed eligible for patients' survey completion and BC-treating physicians were invited to fill the physicians’ questionnaire.

**Results:**

A total of 529 physicians from 50 countries (80 % European) responded, mostly medical oncologists (70 %) working in academic hospitals (53 %). Most physicians request BIS (65 %), mainly when extracranial progression occurs, especially for HER2+ and triple negative BC (TNBC). Among physicians never performing BIS (35 %), 91 % would in case of proved clinical benefit. A total of 545 patients from 14 European countries completed the questionnaire. Median age was 50 years, 86 % had metastatic BC, 51 % hormone receptor-positive (HR+)/HER2-negative, 30 % HER2-positive (HER2+) and 19 % TNBC. BM were diagnosed in 11.5 % patients with metastatic BC. 85 % patients would like to undergo BIS, especially younger ones (p = 0.02) and with HR-disease (p = 0.03), despite the uncertain clinical benefit. Notably, 91 % of patients would like to receive information regarding BM, while only 13 % of physicians routinely address the issue.

**Conclusions:**

These results underline the willingness of patients to know more about the prospects of BM development, in contrast to the lack of routine discussion of this topic by physicians. Further investigation is warranted to demonstrate the clinical utility of routine BIS.

## Highlights

• Most physicians request BIS, in selected cases of MBC, and not as part of routine.
• The majority of patients would like to perform BIS, despite the uncertain benefit.
• The discrepancy between patients desire and physicians initiative stands out.

## 1 Introduction

Breast cancer (BC) is the most frequently diagnosed human malignancy worldwide and one of the leading causes of cancer death in women [1]. Metastatic breast cancer (MBC) is responsible for the vast majority of cancer deaths, remaining an incurable disease [2]. Approximately 5–7 % of breast tumors are diagnosed in the metastatic setting, while roughly 20 % of early breast tumors ultimately develop metastasis at a later stage [3]. Amongst the multiple possible sites of BC metastasis, the brain is possibly the most dreaded. It is estimated that 10–40 % of patients with MBC will develop brain metastases (BM) during the course of disease, depending on the biological subtype of the primary tumor, with increasing incidence observed in the last years [4]. Patients with BM usually experience poor prognosis and impaired quality of life [5]. Therapeutic approaches for BM include surgery, radiotherapy and systemic therapy, variably combined. New targeted therapies arising in the last years are slowly improving patients' prognosis by more effectively targeting metastasis in the brain [[5], [6], [7], [8]]. Still, despite these advances, no routine screening to detect asymptomatic BM is currently recommended for patients with MBC. In fact, it is currently unknown if early detection of BM before becoming symptomatic would translate into better outcomes for patients, either in terms of survival or quality of life improvements. However, discussing brain imaging for early detection of BM may have a rationale, especially in health care environments where a prompt multidisciplinary therapeutic approach would be feasible. However, this additional workup may hold several consequences, including increased costs, exposure to radiation, overdiagnosis and overtreatment. Therefore, incorporating patients’ perspectives in the decision of whether performing or not screening for asymptomatic BM is essential.

With this premise, the “BrainMet BC international” group conducted an international survey aimed at exploring both physicians' and patients’ perspectives regarding routine brain imaging in MBC, to inform the scientific/clinical community and promote a more patient-centered care model on this important topic.

## 2 Methods

### 2.1 Study design

This was an international, cross-sectional, descriptive study, with the primary objective of assessing patients' and physicians' perspectives and attitudes regarding BIS for patients with MBC and no BM diagnosis. As secondary objectives, for the patients' surveys, we aimed to explore the association between clinical-demographic characteristics of patients diagnosed with BC and their willingness to perform brain imaging. As for the physicians' questionnaires, we aimed at evaluating current clinical practices regarding brain imaging assessments and detecting factors that contribute to the decision to request brain imaging in asymptomatic patients with MBC. Finally, we intended to compare patients and physicians’ perspectives regarding routine brain imaging in MBC.

Two questionnaires were developed, the “Brain Imaging in patients with metastatic breast cancer to detect asymptomatic brain metastasis: the physician's perspective” (Q1) (Supplementary material 1) and the “Brain Imaging in patients with metastatic breast cancer to detect asymptomatic brain metastasis: the patients' perspective” (Q2) (Supplementary material 2). Both surveys were designed in English and the patient's perspective survey was subsequently translated in other six different languages (German, French, Dutch, Spanish, Portuguese and Italian). The answers to both questionnaires were drawn up in accordance with a 10-point Likert scale (0-10) or as dichotomous (yes/no). Moreover, questions were added for collection of demographic data and for the characterization of the disease and treatment. Q1 and Q2 comprised 24 and 17 questions, respectively. Both were developed as anonymous questionnaires, requiring 5–7 min to be completed and available as an online survey. Content validation and facial validation of the questionnaire were carried out in two phases. The first phase included the approval by the “BrainMet BC International” group and the second phase required the validation of Q1 by a group of expert physicians, while Q2 was reviewed by 4 different patient advocate groups, from different countries (Germany, Spain, Portugal). An initial pilot test was carried out over the course of 2 months, to assess adherence to the questionnaire and feasibility.

Before filling in Q2, patients were preliminarily informed that there is no general recommendation in international guidelines to perform routine BIS in asymptomatic patients with MBC ([2,9,10]). Patients were also informed that, at present, the clinical benefit associated to earlier detection of asymptomatic BM is unknown.

Individuals considered eligible for data analysis were, for Q1: all physicians treating patients with breast cancer and for Q2: all patients with diagnosis of breast cancer. Q1 was distributed from May 2023 until January 2024 and Q2 from June 2023 until January 2024. The questionnaires were distributed at the responsibility of the “BrainMet BC International” in all countries. Q1 was distributed via social media platforms and newsletters sent to subscribed healthcare professionals through OncoAlert (US and international), GEICAM (Spain), SOLTI (Spain), AIOM (Italy), AGO Mamma and Deutsche Gesellschaft fuer Senologie (Germany), BOOG – Dutch Breast Cancer Research Group (Netherlands). Q2 was distributed mainly through patient advocate groups and patients’ associations, in person and via newsletter. The associations involved were Cancer de Mama Metastásico (Spain), Mamma Mia!, Brustkrebs Deutschland e.V. (Germany), OncoGlam (Portugal) and Dutch Breast cancer association (Netherlands).

Questionnaires were anonymous and data were collected respecting participants’ confidentiality in accordance to national/international regulations. American Association for Public Opinion Research (AAPOR) guidelines were followed. Since the questionnaires were not distributed in clinical or research institutions and were completely anonymous, ethical approval was not required.

### 2.2 Statistical analysis

Data were analyzed from the full analysis population, which included all participants who met inclusion criteria and completed at least 80 % of the survey, to ensure robustness of data. Descriptive analyses of the collected data were performed. Discrete variables were described using absolute and relative frequencies, and continuous variables using trend and dispersion measures. Comparisons within each group were performed using the Mann-Whitney rank sum test for continuous variables and Chi square or Fisher's exact test for categorical variables. Only BC-treating physicians were included. Physicians that request brain imaging in asymptomatic BC patients rarely, sometimes, often or always were categorized as physicians who perform brain imaging in contrast to physicians who stated to never request brain imaging. A logistic regression model was designed to analyze the association between patients' characteristics and willingness to undergo BIS, adjusting for prespecified confounding factors (presence vs absence of diagnosis of MBC; presence vs absence of BM). Statistical analyses were carried out with the Stata IC 15.1 (StataCorp LLC) software.

## 3 Results

### 3.1 Physicians’ survey

**Fig. 1:**
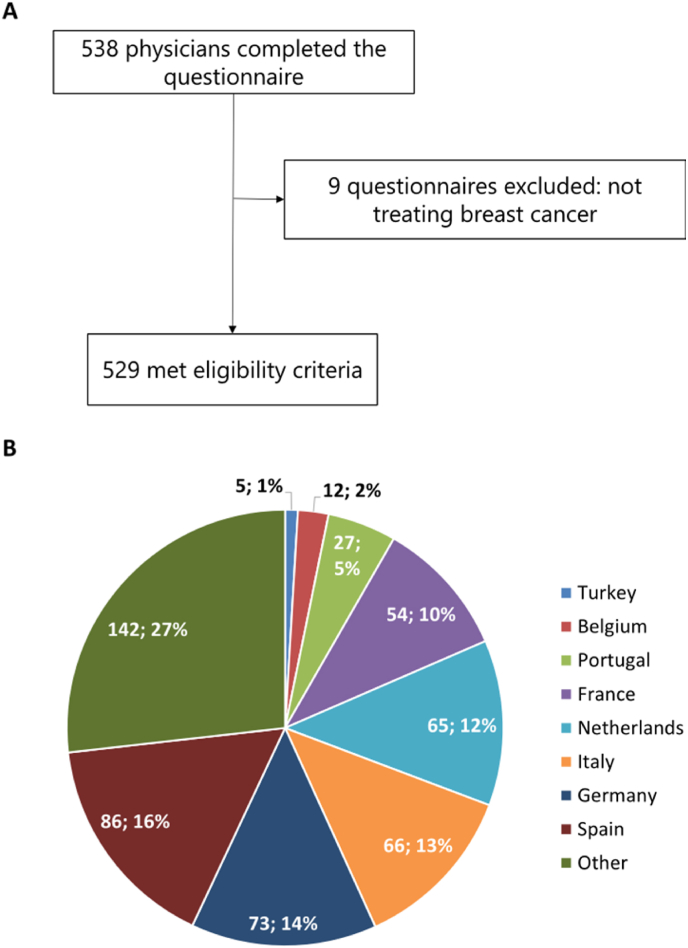
Physicians' inclusion flow-chart and geographical origin of responders **Legend. A:** Physician's survey inclusion flow-chart; **B:** Resonders' geographical origin.

The physician's questionnaire was distributed worldwide. In total, 538 healthcare physicians completed the questionnaire, 9 were excluded because they indicated not to treat BC patients in their daily practice. Hence, a total of 529 met eligibility criteria (Fig. 1A). A total of 50 countries were represented, mostly Spain, Germany, the Netherlands, Italy, Canada, France and Portugal (Fig. 1B).

A total of 329 responders were female (62 %) and 67 % were ≥51 years old. Overall, 91 % responders were specialists, of whom 70 % were medical oncologists, 53 % working for an academic hospital and 14 % in a cancer-specific institution (Table 1).

**Table 1:** Demographic characteristics of responding physicians.

Variable	Overall population	Physicians who routinely request brain imaging	Physicians who do not routinely request brain imaging	p-value
	**N (%)** 529 (100.0)	**N (%)**	**N (%)**	
		346 (65.4)	183 (34.6)	
**Gender, no (%)**				0.779
Male	196 (37.0)	132 (38.1)	64 (35.0)	
Female	329 (62.2)	211 (61.0)	118 (64.5)	
Non-binary	1 (0.2)	1 (0.3)	0 (0)	
Prefer not to specify	3 (0.6)	2 (0.6)	1 (0.5)	
**Age, n (%)**				<0.001
<30	13 (2.5)	9 (2.6)	4 (2.2)	
30-50	162 (30.6)	36 (10.4)	126 (68.8)	
51-60	275 (52.0)	237 (68.5)	38 (20.8)	
>60	79 (14.9)	64 (18.5)	15 (8.2)	
**Medical profession, n (%)**				0.448
Medical oncologist	372 (70.3)	244 (70.5)	128 (69.9)	
Gynecologist	58 (11.0)	42 (12.1)	16 (8.7)	
Radiotherapy oncologist	58 (11.0)	34 (9.8)	24 (13.1)	
Surgeon^a^	29 (5.5)	16 (4.6)	13 (7.1)	
Radiology	5 (0.9)	4 (1.2)	1 (0.6)	
Other^†^	7 (1.3)	6 (1.8)	1 (0.6)	
**Professional position, n (%)**				0.788
Specialist	481 (90.9)	315 (91.0)	166 (90.7)	
Resident/in-training	40 (7.6)	25 (7.3)	15 (8.2)	
Other^‡^	8 (1.5)	6 (1.7)	2 (1.1)	
**Work setting, n (%)**				0.135
Academic hospital	280 (52.9)	184 (53.2)	96 (52.5)	
Non-academic hospital	120 (22.7)	72 (20.8)	48 (26.2)	
Cancer-specific institution	75 (14.2)	47 (13.6)	28 (15.3)	
Private practice	52 (9.8)	41 (11.8)	11 (6.0)	
Other^b^	2 (0.4)	2 (0.6)	0 (0)	

Legend and footnotes.

a Including three neurosurgeons; †three clinical oncologist, two Palliative care physicians, two nurse practitioners; ^‡^Two PhD students, two nurse practitioners, one fellow, one professor, one senior doctor, one head of department;

b One qualifies as/works at an academic hospital, cancer specific institution and private practice as well, one medical care home.

Of the responding physicians, 65 % request BIS in asymptomatic patients with MBC outside the context of a clinical study, while 35 % do not. Most physicians (88 %) are aware of the existence of treatment guidelines for patients with BC and BM, independently of requesting BIS or not (Supplementary Table 1). Demographics, professional position and specialty did not differ between physicians requesting routine BIS or not (Table 1). However, physicians who request brain imaging tended to be older than those who typically do not (p < 0.001) and to be working on European countries (p = 0.001).

Overall, most responders (87 %) stated to only sometimes (70 %) or never (17 %) discuss with their patients the possibility of BM development, while the remaining 13 % discuss it routinely; physicians who request brain imaging routinely do it more regularly, contrarily to physicians who do not request BIS (p < 0.0001) (Supplementary Table 1). Concerning the question on who usually takes the initiative to discuss the topic of BIS for asymptomatic BM detection physicians are the major drivers for these conversations (50 %), followed by patients and their families (25 %), whilst in 18 % of cases no one takes the initiative to approach the issue. Physicians who routinely ask for BIS tend to discuss it more often (p < 0.0001). Notably, 61 % of physicians reported feeling some pressure from patients or their families to perform brain imaging (1 % always, 4 % often, and 57 % sometimes) (Supplementary Table 1).

Among physicians who claimed to never request BIS in asymptomatic patients with MBC (N = 183), the main reason presented was the absence of guideline recommendation (85 %). Other reasons were the potential to increase uncertainties and anxiety (53 %), a potential negative impact on quality of life (39 %), the absence of impact on patients' outcomes (29 %) and costs (21 %) (Supplementary Table 2). We asked these physicians what could change their attitude towards the prescription of routine BIS. Overall, they would request BIS if it would improve patients’ survival (91 %), changed treatment strategy (79 %) or if recommended by international guidelines (57 %) (Table 2). We further questioned if there would be a minimum rate of BM detection by imaging that would make physicians perform BIS and 33 % stated that they would if this rate would be ≥ 30 %. Nonetheless, 27 % kept the statement of not requesting BIS, irrespective of the chance of detecting BM. The decision to request BIS in asymptomatic patients with MBC would be influenced by tumor characteristics for 83 % of physicians not requesting BIS, mainly HER2 positivity (HER2+; 79 %) and in TNBC (63 %) (Supplementary Table 3).

**Table 2:** Reasons that would make physicians willing to request brain imaging in all asymptomatic patients with MBC.

Questionnaire options	Physicians who routinely request brain imaging	Physicians who do not routinely request brain imaging	p-value
	N (%)	N (%)	
	346 (65.4)	183 (34.6)	
**If recommended by international guidelines**			0.0012
No	51 (14.7)	10 (5.5)	
Yes	174 (50.3)	104 (56.8)	
Maybe	91 (26.3)	68 (37.2)	
Missing	30 (8.7)	1 (0.5)	
**If it could change treatment strategy**			0.2202
No	8 (2.3)	7 (3.8)	
Yes	265 (76.6)	144 (78.7)	
Maybe	38 (11.0)	31 (17.0)	
Missing	35 (10.1)	1 (0.5)	
**If it could improve survival of the patient**			0.6427
No	4 (1.2)	3 (1.6)	
Yes	294 (84.9)	167 (91.3)	
Maybe	12 (3.5)	10 (5.5)	
Missing	36 (10.4)	3 (1.6)	
**If it was easily accessible**			<0.0001
No	110 (31.8)	106 (57.9)	
Yes	113 (32.7)	29 (15.9)	
Maybe	89 (25.7)	47 (25.7)	
Missing	34 (9.8)	1 (0.5)	
**Although it could possibly negatively influence quality of life**			0.0004
No	113 (32.7)	78 (42.6)	
Yes	69 (19.9)	15 (8.2)	
Maybe	128 (37.0)	88 (48.1)	
Missing	36 (10.4)	2 (1.1)	
**If the patient asks for it**^a^			
No	–	78 (42.6)	–
Yes	–	21 (11.5)	
Maybe	–	82 (44.8)	
Missing	–	2 (1.1)	

Legend and footnotes.

a This question was only for physicians who answered to not request brain imaging.

Of the 346 (65 %) inquired physicians who request BIS in patients with MBC and no signs of BM, 3 % do it always, 10 % often, 27 % sometimes, and 25 % rarely. A total of 313 physicians justified and gave further information on their attitude towards BIS. The main reason stated was that early diagnosis of metastasis influences patients' quality of life (55 %), followed by BIS having consequences for treatment (50 %) and BIS being suggested by international guidelines (48 %) (Supplementary Table 4). For these physicians, the main reasons to extend brain imaging to all asymptomatic patients with MBC would be the association with improvement in patients' survival (85 %), change in treatment strategy (77 %), the recommendation by international guidelines (50 %) or if it was easily accessible (33 %) (Table 2). To note, 20 % of physicians would request brain imaging despite a possible negative influence on patients’ quality of life. For physicians prescribing BIS, this decision is influenced by BC characteristics for 86 % of inquiries. Specifically, 94 % of physicians would recommend it in HER2+ and 84 % in TNBC (Supplementary Table 3). Of the total inquired physicians who request BIS, 312 answered to questions regarding the timing for brain imaging request: 78 % do it mostly at first diagnosis of MBC and 87 % at time of extracranial progression.

On multivariate regression model building, no clinical-demographic characteristic could predict BIS request (i.e. age, gender, country of practice, institution of practice, specialist vs in training).

### 3.2 Patients’ survey

**Fig. 2:**
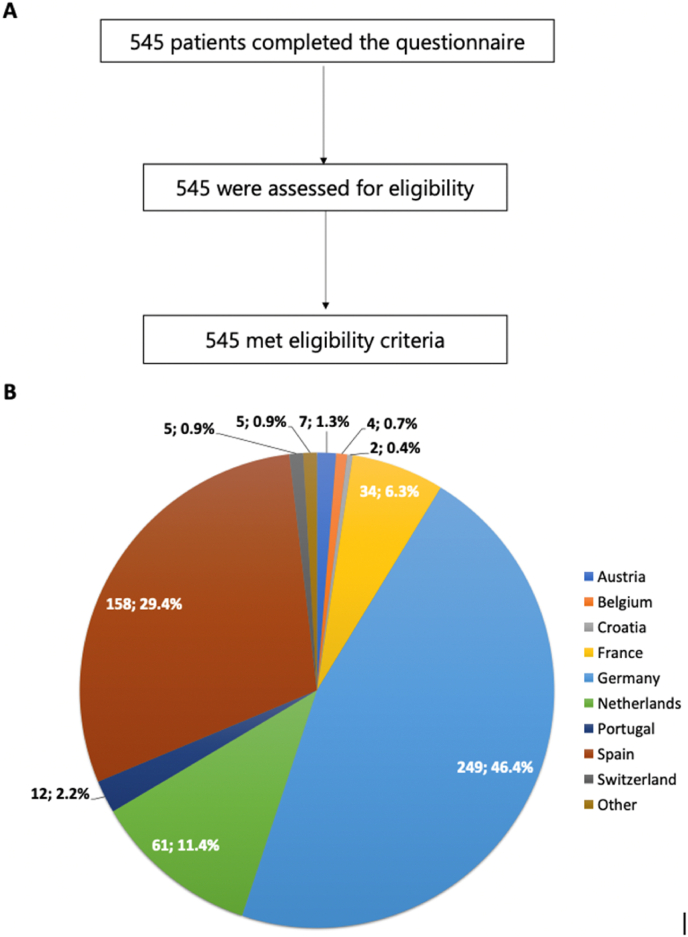
Patients' inclusion flow-chart and geographical origin of responders **Legend. A:** Patient's survey inclusion flow-chart; **B:** Resonders' geographical origin.

The patients’ questionnaire was answered by 545 patients who met eligibility criteria, from 14 different countries (Fig. 2A and 2B). The rate of patients older than 50 years was 50 % and most had high level of education (72 % with university-level degrees of different types). Most patients had a diagnosis of hormone-receptor positive (HR+)/HER2 negative disease (51 %), 30 % HER2+ and 19 % TNBC. A total of 462 (87 %) patients had MBC, and median time since MBC diagnosis was 4 years. A total of 54 (10 %) patients who completed the questionnaire had a diagnosis of BM. Baseline demographic and clinical characteristics of the overall population are summarized in Table 3.

**Table 3:** – Patients’ baseline demographics.

Variable	Overall Population N (%)
	545 (100)
Gender, no (%)	
Female	545 (100)
Age, n (%)	
<30	3 (0.6)
30-50	271 (49.7)
51-60	188 (34.5)
>60	83 (15.2)
Ethnicity, n (%)	
Caucasian	251 (63.5)
Black	6 (1.5)
Hispanic/Latin	12 (3)
Asian	2 (0.5)
Other/Not Reported	124 (31.5)
Level of Education, n (%)	
Bachelor degree or equivalent	216 (40.5)
Doctoral or equivalent level	24 (4.5)
Master's or equivalent level	146 (27.4)
Secondary education	1 (0.2)
Primary Education	26 (4.9)
No scholar education	120 (22.5)
MBC	
Yes, n (%)	462 (85.6)
Median time since MBC diagnosis [IQR], years	4 (2–3)
Brain metastasis, n (%)	54 (9.9)
Breast Cancer biologic subtype	
HR-/HER2+	57 (25.1)
HR+/HER2-	116 (51.1)
HR+/HER2+	12 (5.3)
TNBC	42 (18.5)

**Legend.** MBC, metastatic breast cancer; HR, hormone receptor; HER2, Human Epidermal Growth Factor Receptor 2; TNBC, Triple negative breast cancer; +, positive; -, negative.

**Fig. 3:**
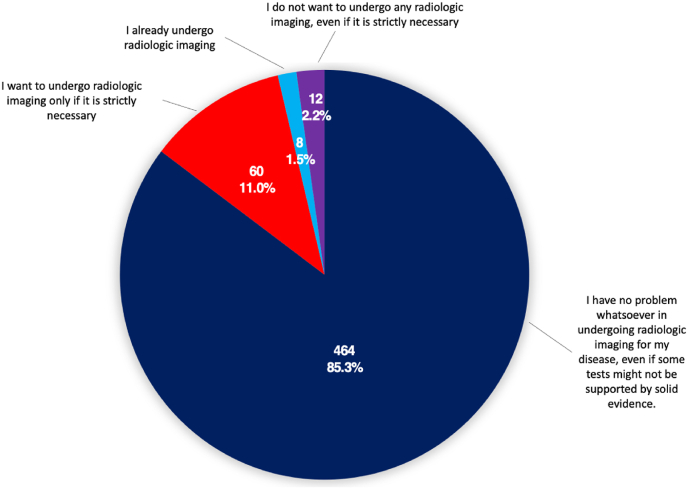
General patients' position regarding imaging exams.

Patients’ general opinion regarding brain radiologic exams was mostly a proactive approach, with 464 (85 %) reporting no specific issue in undergoing radiologic imaging, even if some tests might not be strongly supported by evidence (Fig. 3).

**Fig. 4:**
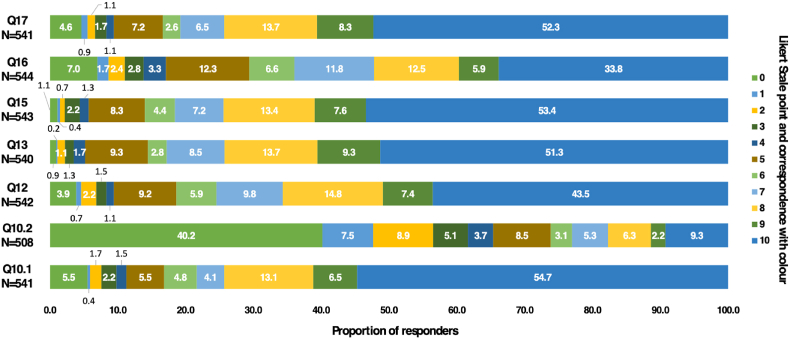
Patient grade of agreement with each sentence/question, according to a 0–10 likert scale.

Regarding patients’ willingness to undergo BIS for asymptomatic BM, 89 % expressed some degree of agreement to perform this screening (punctuation of ≥5 in Likert scale from 0 to 10). The majority of patients (91 %) expressed their desire to discuss the topic more extensively with their physician (punctuation of ≥5 in Likert scale from 0 to 10) and 83 % expressed interest in participating in clinical trials, including with experimental drugs (punctuation of ≥5 in Likert scale from 0 to 10). Accordingly, 95 % of patients expressed their willingness to undergo invasive local treatments (e.g. surgery or radiotherapy) in case of BM diagnosis (punctuation of ≥5 in Likert scale from 0 to 10). Also, most patients (91 % with punctuation of ≥5 in Likert scale from 0 to 10) would participate in observational studies to address the benefit to perform BIS (Fig. 4).

To note, 65 % of patients stated to have never discussed performing periodical imaging exams to the brain with their physician, but 48 % reported to have searched for information on their own concerning this topic, either on the internet or other sources. Notably, 86 % of patients would like to be informed in the event of BM.

Furthermore, 35 % of responders showed no interest to undergo BIS in the absence of proven benefit (punctuation of ≥5 in Likert scale from 0 to 10) (Fig. 4). Using a multivariable logistic regression model to identify predictors of patients’ willingness to undergo BIS and adjusting for diagnosis of MBC, presence of BM and level of education, odds were lower with increasing age (OR 0.97, 95 % CI 0.95–0.99 p = 0.020) and a diagnosis of HR + disease (OR 0.40, 95 % CI 0.17–0.93, p = 0.032) (Supplementary Table 5).

## 4 Discussion

We conducted an international cross-sectional study to investigate BC-treating physicians' and BC patients' perspectives on routine BIS to detect asymptomatic BM in patients with MBC. An online survey was distributed and answered by 529 physicians and 545 patients from several different countries. The vast majority of physicians was aware of the existence of treatment guidelines for patients with MBC with brain involvement; nonetheless, despite international guidelines do not generally recommend routine BIS for asymptomatic patients, most physicians stated to request brain imaging (65 %) in asymptomatic patients. This was especially true among older clinicians. Namely, 87 % of physicians usually requesting BIS were older than 50, while among physicians not requesting BIS on a routine basis, 71 % were younger than 50. This striking contrast might be explained by a generational difference related to the worldwide spreading of Evidence-Based Medicine in the last decades and a more guideline-centered approach adopted by clinicians with less practical experience. Importantly, recommendation by international guidelines, implications for treatment and impact on survival were the most relevant causes for a potential change in physicians' attitude for both responders' groups. Noteworthy, physicians usually requesting BIS were more likely to do so irrespective of a potential negative effect on patients' quality of life than physicians who do not usually perform a BIS. This attitude was consistent with the evidence of certain lack of information reported by patients in their questionnaire and might reflect a paternalistic attitude that was widespread among physicians in previous decades [11]. This data would thus be coherent with physicians usually requesting BIS being older than those who usually do not. Additionally, we observed that physicians working in non-European countries were less likely to request BIS. The representation of these countries was vast and included low and middle-income countries (e.g. Morocco, Iraq, Cambodia) and these results may be explained by the lack of easy access to imaging exams, reimbursement constraints and country-level differences regarding available treatment options. Nevertheless, we hereby highlight the need to account for patients’ preferences, views and perspectives, prioritizing patient-centered care and quality of life, especially imperative in the context of an incurable metastatic disease.

Regarding patients, the broad availability to undergo BIS, irrespective of the presence of symptoms and without evidence of gaining any meaningful benefit from the procedure, was associated with a prominent willingness (91 % of patients) to be more informed about the topic of BM by their treating physicians. This perspective was in striking contrast with 87 % of physicians never or only sometimes engaging their patients in a discussion on the issue of potential BM development during the natural course of the disease. The perspective emerging from this survey is important and confirms the willingness of most patients with BC to receive adequate information regarding their disease. Still, there is also a proportion of patients who did not express the will to receive more information on this topic in line with other studies highlighting that not all oncologic patients want to receive all the information available [12]. A personalized patient-centered approach focused on understanding each single patient's needs appear to be the most adequate solution.

The majority of responding physicians was clearly aware of the highest risk of BM development in HER2+ and TNBC [9], as these biologic subtypes were those for which physicians appeared to be more eager to request brain imaging assessment. Also, amongst patients, a diagnosis of HR + disease was an independent predictor of less willingness to undergo brain imaging exams. This demonstrates some degree of health literacy among interviewed patients, in line with previous reports [20]. Notably, approximately 50 % of physicians who do not routinely request BIS would consider a detection rate of 30 % or even 10 % for BM as the minimum threshold to justify prescribing BIS in asymptomatic patients. The recently published results of the phase II trial of brain surveillance in patients with MBC by Ahmed et al., showed considerable cumulative rates of asymptomatic BM in patients with HER2+ and TNBC (24 and 25 %, respectively) [13]. Moreover, 16 % (yes) to 26 % (maybe) of these physicians would change their approach if access to brain imaging was improved. Together, these data strengthen the need to dissect whether early diagnosis of BM can translate into better outcomes for patients, while engaging them into shared decision-making regarding BIS. In this perspective, several working groups are performing randomized clinical trials evaluating the role of BIS (e.g. NCT04030507). If these trials succeed, the potential rise in healthcare costs that could derive by an increased BIS utilization, without guaranteed patient benefits, underscores the critical need to invest in the development of effective therapeutic strategies for BM, as well. These strategies should aim to enhance patient outcomes, by improving survival rates and/or quality of life.

Importantly, physicians and patients appear to be aligned in their views. More than 80 % of responding patients indicated a willingness to participate in observational or interventional clinical trials with new treatments in this area. Simultaneously, a majority of physicians are awaiting evidence of a survival benefit and change in guideline recommendations, before modifying their practices. This alignment between key stakeholders provides a fertile land for further research on this topic. At the same time, current trials are underway (NCT04030507, NCT03881605, NCT05115474) [[14], [15], [16]], despite challenges in meeting recruitment expectations. This might be due to the randomized trial design and unwillingness of the patients to be randomized in the control cohort. Hence, different approaches should be envisioned to facilitate and maximize recruitment, while still retaining the power to answer clinically meaningful questions.

Addressing the treatment of BM remains an unmet need, given the poor prognosis associated with its presence. Therefore, efforts should focus on developing novel treatments capable of crossing the blood-brain barrier to provide greater therapeutic efficacy or improve the efficacy of locoregional treatments and their tolerability [17]. Recent advancements have been made with systemic drugs like trastuzumab deruxtecan in HER2+ and HER2-low MBC, sacituzumab govitecan in HR+/HER2-negative and TNBC, and tucatinib-based treatment in HER2+ MBC [[18], [19], [20], [21], [22], [23]] as well as CDK4/6 inhibitor-based treatment in HR+/HER2-disease [24]. However, there is considerable room for improvement, especially beyond HER2+ disease. An interesting approach under clinical investigation is the development of therapeutic strategies to prevent the development of BM (e.g. low-dose temozolomide + T-DM1 or tucatinib based therapy in HER2+ MBC [25].

Furthermore, artificial intelligence (AI) tools may refine our ability to decide which patients with MBC should undergo BIS, by leveraging advanced data analysis, predictive modelling and personalized risk assessment. Clinical decision support systems, integrating clinical, pathological and imaging data, together with molecular profiling can enhance screening guidelines for different patient groups [26].

Our study is not exempt from limitations. First, we have developed two questionnaires that had not been previously validated elsewhere. Responders to both questionnaires proceeded from many different countries, with some countries providing only 1–2 responses, restraining more in deep analysis of regional difference. Another limitation was that responding physicians were not only medical oncologists and gynecologists, who would be the most logical physicians to request brain imaging for their patients; nonetheless, we only analyzed the answers of physician who declared to treat patients with BC in their daily practice. Finally, regarding Q2, a potential selection bias should be acknowledged, i.e. patients who decided to take the survey could be those more interested/touched by the topic, and the sample might be not fully representative of the entire patient population.

In any case, this is the first study to provide simultaneous insight into physicians and patients perspective on the topic of asymptomatic BIS, and is also the first to address this on an international level, collecting opinions from all over the globe. Both health professionals and patients' advocates were directly involved in the questionnaires’ development, assuring a broad range of different skills, perspectives and sensitivities and strengthening the relevance of the surveys.

## 5 Conclusion

This study sheds lights on physicians' perspectives and patients’ preferences, regarding a delicate topic that, while still encompassing a dismal prognosis, has witnessed deep improvements in treatment approaches in the last few years. The results provide a solid ground for further investigations to assess the actual clinical value of BIS in patients with MBC, as well as the development of more effective tools to enhance physician-patient communication and shared decision-making. AI-driven tools to leverage real-world data may represent a step forward, enabling the development of instruments capable of providing individualized, precise, and timely screening, diagnostics, and treatment options, ultimately leading to measurable benefits for patients, healthcare systems, and society as a whole.

## CRediT authorship contribution statement

**Leonor Matos:** Conceptualization, Data curation, Formal analysis, Investigation, Methodology, Project administration, Resources, Software, Supervision, Validation, Visualization, Writing – original draft, Writing – review & editing. **Mette van Ramshorst:** Conceptualization, Formal analysis, Methodology, Writing – original draft. **Volkmar Müller:** Conceptualization, Methodology, Writing – review & editing. **Elisa Agostinetto:** Conceptualization, Data curation, Investigation, Methodology, Writing – review & editing. **Sabine Linn:** Methodology, Supervision, Writing – review & editing. **Matteo Lambertini:** Visualization, Writing – review & editing. **Veronique Dieras:** Methodology, Writing – review & editing. **Fanny le Du:** Data curation, Writing – review & editing. **Sofia Braga:** Writing – review & editing. **Carmen Criscitiello:** Validation, Visualization, Writing – review & editing. **Katarzyna J. Jerzak:** Supervision, Validation, Writing – review & editing. **Gil Morgan:** Data curation, Resources, Software, Validation. **Sara Brucker:** Validation, Visualization, Writing – review & editing. **Patricia von Kroge:** Data curation, Resources, Validation, Visualization, Writing – review & editing. **Renate Haidinger:** Resources, Validation, Conceptualization. **Gema Rodríguez Recio:** Validation, Visualization, Writing – review & editing, Conceptualization. **Eva Schumacher-Wulf:** Conceptualization, Data curation, Supervision, Validation, Visualization, Writing – review & editing. **Mario Fontes Sousa:** Conceptualization, Data curation, Formal analysis, Investigation, Methodology, Validation, Writing – original draft, Writing – review & editing. **Francesco Schettini:** Conceptualization, Data curation, Formal analysis, Investigation, Project administration, Resources, Supervision, Validation, Writing – original draft, Writing – review & editing. **Elena Laakmann:** Conceptualization, Data curation, Formal analysis, Investigation, Methodology, Project administration, Resources, Supervision, Validation, Visualization, Writing – original draft, Writing – review & editing.

## Data sharing statement

The empty questionnaires are available as **Supplementary Materials.** Despite being anonymized, to protect responders′ privacy, results can be accessed only upon reasonable request to the corresponding authors and posterior approval by all authors after careful evaluation of the request.

## Funding

This work received no funding.

## Declaration of competing interest

LM reports Speaker/Consultancy fee: Novartis, Daiichi Sankyo, AstraZeneca, Lilly, Pfizer, Roche. Support for attending medical conferences from: Novartis, Roche, Pfizer, Lilly. EA reports Advisory board or honoraria from: Eli Lilly, AstraZeneca, Abscint, Bayer. Research grant to my Institution from Gilead. Travel grants/expenses from: Novartis, Eli Lilly, Daiichi Sankyo, AstraZeneca, Abscint, Menarini (all outside the submitted work). FS reports honoraria from Novartis, Gilead, Veracyte and Daiichy-Sankyo for educational events/materials, travel expenses from Novartis, Gilead and Daiichy-Sankyo and advisory board fees from Pfizer and Veracyte. EL reports travel expenses from Pierre Fabre, Astra Zeneca and Daiicho Sankyo honoraria for educational events from Astra Zeneca, Seagen, Gilead and Pierre Fabre and advisory board fees from Novartis, Astra Zeneca, Gilead and Daiichi Sankyo (all outside this manuscript). Given their role as [Speciality Editor], [Volkmar Müller] had no involvement in the peer-review of this article and has no access to information regarding its peer-review. Full responsibility for the editorial process for this article was delegated to another journal editor. VM reports Speaker honoraria: Astra Zeneca, arsTempi, Daiichi-Sankyo, Eisai, Pfizer, MSD, Medac, Novartis, Roche, Seagen, Onkowissen, high5 Oncology, Lilly, Medscape, Gilead, Pierre Fabre, iMED Institute. Consultancy honoraria: Roche, Pierre Fabre, PINK, ClinSol, Novartis, MSD, Daiichi-Sankyo, Eisai, Lilly, Seagen, Gilead, Stemline.

Institutional research support: Novartis, Roche, Seagen, Genentech, Astra Zeneca. Travel grants: Astra Zeneca, Roche, Pfizer, Daiichi Sankyo, Gilea. The other authors report no conflict of interest.
