# Supplementary material for: Brain imaging screening in metastatic breast cancer: patients’ and physicians’ perspectives
Source: Breast. 2025 Aug 12;84:104558. doi: 10.1016/j.breast.2025.104558 (PMC12683136; doi:10.1016/j.breast.2025.104558)
Supplement: Multimedia component 1 [file mmc1.docx]

**Supplementary materials**

1. Physicians’ questionnaire: “Brain Imaging in patients with metastatic breast cancer to detect asymptomatic brain metastasis: the physician’s perspective” 2

2. Patients’ questionnaire: “Brain Imaging in patients with metastatic breast cancer to detect asymptomatic brain metastasis: the patients’ perspective” 8

3. Supplementary tables 11

Supplementary table 1. Overall questions to physicians regarding brain imaging for asymptomatic patients with metastatic breast cancer 11

Supplementary table 2. Reasons for physicians not to perform brain imaging in asymptomatic patients with metastatic breast cancer 12

Supplementary table 3. Breast cancer characteristics that would make physicians more willing to perform brain imaging in asymptomatic breast cancer patients 13

Supplementary table 4. Reasons for physicians to perform brain imaging in asymptomatic patients with breast cancer 14

Supplementary table 5. Multivariate logistic regression for the outcome willingness to undergo brain imaging screening 15

# **1. Physicians’ questionnaire: “Brain Imaging in patients with metastatic breast cancer to detect asymptomatic brain metastasis: the physician’s perspective”**

**Brain imaging in patients with metastatic breast cancer to detect asymptomatic brain metastases: the physician’s perspective.**

**Time investment:** approximately 5-7 minutes

**Rationale:** Due to significant therapeutic improvements, patients with metastatic breast cancer live longer. With more years to live, incidence of brain metastases has also increased. Currently, it is not recommended to request brain imaging in patients with metastatic disease without neurological symptoms. In fact, there is no data supporting that an anticipated diagnosis of asymptomatic brain metastases can have a positive impact on patient survival and quality of life. Considering the current availability of potentially effective treatment options, including novel systemic treatments, stereotactic radiotherapy and refined neurosurgical procedures, the detection of asymptomatic brain metastases could lead physicians to change therapeutic strategy. This could potentially benefit patients, but at the same time, it might lead to overtreatment, with relevant implications in terms of patient care costs and risks for adverse events.

**Aim of our research project:** We would like to explore physicians’ opinion regarding routine brain imaging in metastatic breast cancer patients without symptoms related to brain metastases.

**Future perspective:** Your opinion may help define recommendations about brain imaging in asymptomatic patients and/or help design a prospective clinical trial to further explore this topic.

**Questions:**

1. Do you treat patients with breast cancer in your daily activity?
   1. Yes
   2. No

*If answered with b (no) this questionnaire does not apply to you and you can stop here.*

1. Country where you practice (drop-down list)
2. Your age
   1. <30
   2. 30-50
   3. 50-60
   4. >60
3. Gender
   1. Male
   2. Female
   3. Non binary
   4. Prefer not to specify
4. Specialty/profession
   1. Medical Oncologist
   2. Gynecologist
   3. Radiologist
   4. Neurosurgeon
   5. Surgeon
   6. Radiotherapist
   7. Other (please specify)
5. Professional situation
   1. Resident/in-training
   2. Specialist
   3. Other (please specify)
6. Work setting
   1. Academic hospital
   2. Non-academic hospital
   3. Private practice/hospital
   4. Cancer-specific Institution
   5. Other (please specify)
7. Are you aware of the existence of treatment guidelines for patients with breast cancer and brain metastases?
   1. No
   2. Yes
8. Do you routinely talk about the possibility of brain metastases in asymptomatic metastatic breast cancer patients?
   1. Never
   2. Sometimes
   3. Always
9. Who usually takes the initiative to talk about brain imaging for detection of asymptomatic brain metastases during consultation (more answers are possible)?
   1. Me (the physician)
   2. The patient or his/her family/caregivers
   3. No one
   4. Not applicable
10. Do you feel pressure to request brain imaging by your patient or his/her caregivers?
    1. Never
    2. Sometimes
    3. Often
    4. Always
11. Would you support the idea of submitting a similar questionnaire to patients, to explore their opinion on this topic?
    1. Absolutely yes
    2. Yes, but only in metastatic patients
    3. Yes, but only in patient with brain metastases
    4. Absolutely no
    5. I don’t know
    6. Other (please specify)

Please provide an explanation for your answer (optional):

1. Do you request brain imaging in metastatic breast cancer patients with no symptoms of brain metastases?
   1. Never
   2. Rarely
   3. Sometimes
   4. Often
   5. Always

*Q13. If answered with a go to part* ***A***

*Q13. If answered with b or c or d or e go to part* ***B***

***Please fill in A or B***

***MANDATORY until Question 13***

**A**

1. You do not request brain imaging in asymptomatic metastatic breast cancer patients because (more answers possible):
   1. it is not recommended by guidelines
   2. to avoid radiation exposure
   3. of costs
   4. of long waiting list for imaging/limited availability
   5. it does not change the outcome of my patient
   6. it does not have consequences for the treatment
   7. it creates more uncertainties and anxiety
   8. early diagnosis of asymptomatic brain metastases may negatively influence the quality of life of my patient
   9. the patient is treated with agents that cross the blood-brain barrier anyway
   10. the patient is treated with agents that do not cross the blood-brain barrier
   11. of other reasons (please describe)
2. Would you request brain imaging in asymptomatic patients if it was recommended by international guidelines?
   1. No
   2. Yes
   3. Maybe
3. Would you request brain imaging in asymptomatic patients if it could change treatment strategy?
   1. No
   2. Yes
   3. Maybe
4. Would you request brain imaging in asymptomatic patients if it would improve survival of the patient?
   1. No
   2. Yes
   3. Maybe
5. Would you request brain imaging in asymptomatic patients although it could negatively influence quality of life?
   1. No
   2. Yes
   3. Maybe
6. Would you request brain imaging in asymptomatic patients if it was easily accessible?
   1. No
   2. Yes
   3. Maybe
7. Would you request brain imaging in asymptomatic patients if the patient asks for it?
   1. No
   2. Yes
   3. Maybe
8. Would breast cancer subtype or tumor biology influence your decision to request brain imaging in asymptomatic patients?
   1. No
   2. Yes
   3. Maybe
9. Which breast cancer characteristics would make you more willing to request brain imaging in asymptomatic patients (select all that apply):
   1. None (I will not request it regardless of characteristics)
   2. Triple negative
   3. Luminal A-like
   4. Luminal B-like
   5. HER2-positive
   6. Lobular carcinoma
   7. Ductal carcinoma
   8. High Ki-67
   9. High tumor grade
   10. Other (please describe)
10. I would request brain imaging in asymptomatic patients if the chance of detecting brain metastasis would be:
    1. irrespective of the chance of detecting I would not request it
    2. ≥10%
    3. ≥30%
    4. ≥50%
    5. ≥70%
    6. irrespective of the chance of detecting I would request it
    7. other (please specify)

**B**

1. You do request brain imaging in asymptomatic metastatic breast cancer patients, because (more answers possible):
   1. guidelines provide general indications, but some clinical cases might require a different management
   2. it is cheap
   3. it is easily accessible
   4. it may improve the outcome of the patient
   5. it has consequences for the treatment
   6. early diagnosis of asymptomatic brain metastases influences the quality of life of the patient
   7. it diminishes uncertainties and anxiety
   8. the patient requested it
   9. for advanced care planning
   10. of other reasons (please describe)
2. Would you request brain imaging in asymptomatic patients if it was recommended by international guidelines?
   1. No
   2. Yes
   3. Maybe
3. Would you request brain imaging in asymptomatic patients if it could change treatment strategy?
   1. No
   2. Yes
   3. Maybe
4. Would you request brain imaging in asymptomatic patients if it would improve survival of the patient?
   1. No
   2. Yes
   3. Maybe
5. Would you request brain imaging in asymptomatic patients although it could negatively influence quality of life?
   1. No
   2. Yes
   3. Maybe
6. Would you request brain imaging in asymptomatic patients if it was easily accessible?
   1. No
   2. Yes
   3. Maybe
7. Timing of brain imaging: do you request brain imaging in asymptomatic patients for staging at first diagnosis of metastatic disease?
   1. No
   2. Sometimes
   3. Often
   4. Always
8. Timing of brain imaging: do you request brain imaging in asymptomatic patients at time of progression of extracranial disease?
   1. No
   2. Sometimes
   3. Often
   4. Always
9. Does the subtype or tumor biology of breast cancer influence the decision to request brain imaging in asymptomatic patients?
   1. No
   2. Sometimes
   3. Often
   4. Always
10. If the previous question was answered with b (sometimes) or c (often) or d (yes): in which subtype are you more willing to request brain imaging in asymptomatic patients? (select all that apply)
    1. Triple negative
    2. Luminal A-like
    3. Luminal B-like
    4. HER2-positive
    5. Lobular carcinoma
    6. Ductal carcinoma
    7. High Ki-67
    8. High tumor grade
    9. Other (please describe)
11. I would request brain imaging in asymptomatic patients if the chance of detecting brain metastasis would be:
    1. irrespective of the chance of detecting I would not request it
    2. ≥10%
    3. ≥30%
    4. ≥50%
    5. ≥70%
    6. irrespective of the chance of detecting I would request it
    7. other (please specify)

# **2. Patients’ questionnaire: “Brain Imaging in patients with metastatic breast cancer to detect asymptomatic brain metastasis: the patients’ perspective”**

**PATIENTS AND PHYSICIANS PERSPECTIVES ON BRAIN IMAGING FOR PATIENTS WITH METASTATIC BREAST CANCER**

**Please answer to this survey only once.**

We ask you to carefully read the following information.

In patients with a diagnosis of metastatic breast cancer, there is currently no evidence to routinely recommend imaging exams of the brain in the absence of neurologic or cognitive signs or symptoms that may suggest brain metastasis. Our question is: *Should we be performing routine imaging to the brain in patients with metastatic breast cancer in the absence of symptoms of brain involvement?* This survey intends to better understand your perception and perspective on this subject, in order to better answer this question and meet your preferences, aiming at improving the quality of breast cancer care. The researchers assure the anonymity and confidentiality of all data.

1. **Age** __________ (Years)
2. **Nationality (country where you have lived most of your life)** ________
3. **Education:**

No schooling ◻ Primary Education ◻ Secondary education ◻ Bachelor degree or equivalent ◻
Master’s or equivalent level ◻ Doctoral or equivalent level ◻

1. **Do you have a diagnosis of breast cancer with metastasis (stage IV breast cancer)?**

YES ◻ NO ◻

If you answered NO to this question, you have finished this survey. Thank you for your participation.

1. **When were you diagnosed with metastasis from breast cancer?** _________ (year)
2. **According to your physician and passed exams, do you have brain metastasis from breast cancer?**

YES ◻ NO ◻ DO NOT KNOW ◻

1. **Are you currently doing treatments for your cancer?**

YES, CHEMOTHERAPY ◻ YES, HORMONE THERAPY ◻ YES, TARGETED THERAPY ◻ NO ◻

1. **Would you be willing/feel comfortable to perform periodic imaging exams to the brain, in the absence of symptoms or signs that raise a suspicion for brain metastasis, even without existing evidence that this is beneficial for you?**

LIKERT SCALE

1. **Which is your attitude towards imaging tests?**

◻ I have no problem whatsoever in undergoing radiologic imaging for my disease, even if some tests might not be supported by solid evidence;

◻ I want to undergo radiologic imaging only if it is strictly necessary;

◻ I already undergo radiologic imaging;

◻ I do not want to undergo any radiologic imaging, even if it is strictly necessary

1. **Please indicate how much you agree with the following statements:**
   1. I would like to undergo an imaging exam of my brain to exclude the presence of any metastases, also in absence of any symptoms;

LIKERT SCALE

- 1. I would not like to undergo an imaging exam of my brain, if there is no proven benefit that can derive from it;

LIKERT SCALE

1. **Have you ever discussed performing periodical imaging exams to the brain with your physician?**

NO ◻ YES ◻

1. **Would you like to discuss this topic more extensively with your physician?**

LIKERT SCALE

1. **In case brain metastasis would be detected, would you be comfortable with performing invasive procedures?**

LIKERT SCALE

1. **Have you ever searched for this topic on the internet or other sources?**

NO ◻ YES ◻

1. **Would you like to know more about this topic?**

LIKERT SCALE

1. **Would you be willing to participate in a trial that would help to provide an answer to this question?**

LIKERT SCALE

1. **Would you be willing to participate in a purely observational study (meaning without experimental treatments) designed to address these questions?**

LIKERT SCALE

PLEASE VERIFY IF YOU HAVE ANSWERED TO ALL QUESTIONS

Thank you very much for you time

# **3. Supplementary tables**

## **Supplementary table 1. Overall questions to physicians regarding brain imaging for asymptomatic patients with metastatic breast cancer**

| **Questionnaire options** | **Overall Population** | | | **Physicians who routinely request brain imaging** | | | **Physicians who do not routinely request brain imaging** | | | | **p-value** |  |
| --- | --- | --- | --- | --- | --- | --- | --- | --- | --- | --- | --- | --- |
|  | **N** | | **%** | **N** | **%** | **N** | | **%** | |  | | |
|  | 529 | | 100 | 346 | 65.4 | 183 | | 34.6 | |  |  |  |
| **Are you aware of the existence of treatment guidelines for patients with breast cancer and brain metastases?** | | | | | | | | | | | 0.328 |  |
| No | 65 | | 12.3 | 39 | 11.2 | 26 | | 14.2 | |  | | |
| Yes | 464 | 87.7 | | 307 | 88.8 | 157 | | 85.8 | |  |  |  |
| **Do you routinely talk about the possibility of brain metastases in asymptomatic metastatic breast cancer patients?** | | | | | | | | | | <0.0001 | | |
| Never | 91 | 17.2 | | 20 | 5.7 | 71 | | 38.8 | |  | | |
| Sometimes | 370 | 69.9 | | 262 | 75.7 | 108 | | 59.0 | |  |  |  |
| Always | 68 | 12.9 | | 64 | 18.6 | 4 | | 2.2 | |  |  |  |
| **Who usually takes the initiative to talk about brain imaging for detection of asymptomatic brain metastases during consultation (more answers are possible)** | | | | | | | | | | <0.0001 | | |
| The physician | 263 | 49.7 | | 227 | 65.7 | 36 | | 19.7 | |  | | |
| The patient or his/her family | 131 | 24.7 | | 77 | 22.3 | 54 | | 29.5 | |  |  |  |
| No one | 95 | 17.9 | | 32 | 9.2 | 63 | | 34.4 | |  |  |  |
| Not applicable | 40 | 7.7 | | 10 | 2.8 | 30 | | 16.4 | |  |  |  |
| **Do you feel pressure to request brain imaging by your patient or his/her caregivers?** | | | | | | | |  | <0.0001 | | | |
| Never | 207 | 39.1 | | 103 | 29.8 | 104 | | 56.8 | |  | | |
| Sometimes | 300 | 56.7 | | 225 | 65.0 | 75 | | 40.4 | |  |  |  |
| Often | 19 | 3.6 | | 16 | 4.6 | 3 | | 1.5 | |  |  |  |
| Always | 3 | 0.6 | | 2 | 0.6 | 1 | | 0.5 | |  |  |  |
| **Would you support the idea of submitting a similar questionnaire to patients, to explore their opinion on this topic?** | | | | | | | | | | 0.047 | | |
| Yes | 176 | 33.3 | | 130 | 37.6 | 46 | | 25.1 | |  | | |
| Only in metastatic patients | 159 | 30.1 | | 100 | 28.9 | 59 | | 32.3 | |  |  |  |
| Only in patient with brain metastases | 32 | 6.0 | | 18 | 5.2 | 14 | | 7.7 | |  |  |  |
| No | 48 | 9.1 | | 25 | 7.1 | 23 | | 12.6 | |  |  |  |
| I don’t know | 98 | 18.5 | | 63 | 18.1 | 36 | | 19.6 | |  | | |
| Other | 16 | 3.0 | | 11 | 3.1 | 5 | | 2.7 | |  | | |

## **Supplementary table 2. Reasons for physicians not to perform brain imaging in asymptomatic patients with metastatic breast cancer**

|  | **Total N=183** | |
| --- | --- | --- |
|  | **N** | **%*** |
| It is not recommended by the guideline | 154 | 84.6 |
| It creates more uncertainties and anxiety | 96 | 52.7 |
| Early diagnosis of metastasis may negatively influence quality of life | 71 | 39.0 |
| It does not change the outcome of the patient | 53 | 29.1 |
| Costs | 38 | 20.9 |
| No consequences for the treatment | 35 | 19.2 |
| Long waiting lists/limited availability | 29 | 15.9 |
| To avoid radiation exposure | 19 | 10.4 |
| Patients is treated with agents that cross the blood-brain barrier anyway | 10 | 5.5 |
| Patient is treated with agents that do not cross the blood-brain barrier | 2 | 1.1 |
| Other reasons | 13 | 7.1 |
| Missing^#^ | 1 | 0.5 |

**Legend.***Percentages do not sum up to 100% as more than 1 answer option was allowed and are calculated excluding missings. # the proportion of missing is calculated on the total number of patients/group.

## **Supplementary table 3. Breast cancer characteristics that would make physicians more willing to perform brain imaging in asymptomatic breast cancer patients**

|  | **Physicians who do not request imaging** | | **Physicians who**  **do request  imaging** | |
| --- | --- | --- | --- | --- |
|  | **Total N = 183** | | **Total N = 346** | |
|  | **N** | **%*** | **N** | **%*** |
| HER2-positive breast cancer | 145 | 79.2 | 293 | 93.9 |
| Triple negative breast cancer | 116 | 63.4 | 261 | 83.7 |
| High tumor grade | 21 | 11.5 | 61 | 19.6 |
| High Ki67 | 19 | 10.4 | 69 | 22.1 |
| Luminal B-like breast cancer | 6 | 3.3 | 29 | 9.3 |
| Luminal A-like breast cancer | 1 | 0.5 | 4 | 1,3 |
| Lobular carcinoma | 5 | 2.7 | 25 | 8.0 |
| Ductal carcinoma | 1 | 0.5 | 6 | 1.9 |
| None, will not request it regardless of characteristics | 27 | 14.8 | - | - |
| Other reasons | 2 | 1.1 | 6 | 1.9 |
| Missing^#^ | 1 | 0.5 | 32 | 9.2 |

**Legend.** *Percentages do not sum up to 100% as more than 1 answer option was allowed and are calculated excluding missings. # the proportion of missing is calculated on the total number of patients/group.

## **Supplementary table 4. Reasons for physicians to perform brain imaging in asymptomatic patients with breast cancer**

|  | **Total responders N=313** | |
| --- | --- | --- |
|  | **N** | **%*** |
| Early diagnosis of metastasis influences the quality of life of the patient | 172 | 55.0 |
| It has consequences for the treatment | 155 | 49.5 |
| It is suggested as an option in the guidelines | 149 | 47.6 |
| It may improve the outcome of the patient | 117 | 37.4 |
| For advanced care planning | 64 | 20.4 |
| It diminishes uncertainties and anxiety | 58 | 18.5 |
| The patient requested it | 58 | 18.5 |
| It is easily accessible | 46 | 14.7 |
| It is cheap | 6 | 1.9 |
| Other reasons | 18 | 5.8 |
| Missing^#^ | 33 | 9.5 |

**Legend.** *Percentages do not sum up to 100% as more than 1 answer option was allowed and are calculated excluding missings. # the proportion of missing is calculated on the total number of patients/group.

## **Supplementary table 5. Multivariate logistic regression for the outcome willingness to undergo brain imaging screening**

| **VARIABLES** | **MULTIVARIATE ANALYSIS** | | |
| --- | --- | --- | --- |
|  | **Odds Ratio** | **95% CI** | ***p*-value** |
| Age | 0.97 | 0.95-0.99 | 0.020* |
| Level of Education | 1.05 | 0.67-1.63 | 0.817 |
| Diagnosis of MBC | 2.45 | 0.88-7.07 | 0.084 |
| Diagnosis of BM | - | - | - |
| HR+ BC | 0.40 | 0.17-0.93 | 0.032* |
| HER2+ BC | 0.6 | 0.23-1.54 | 0.291 |

***All p-values < 0.05 in univariate analysis were used in the multivariate model.***

***(*-statistically significant values).***

Age was included as continuous variable. Level of Education was included as a categorical variable.

MBC, metastatic breast cancer; BM, brain metastases, HR+ - hormone receptor positive; BC, Breast Cancer, HER2, Human epidermal growth factor receptor-2.
